# Provider Training in the Management of Headache Following Concussion Clinical Recommendation: Promoting a Standardized Means for Efficient Patient Recovery and Timely Return to Duty

**DOI:** 10.3389/fneur.2020.559311

**Published:** 2020-10-15

**Authors:** Rosemay A. Remigio-Baker, Seth Kiser, Hamid Ferdosi, Emma Gregory, Scot Engel, Sean Sebesta, Daniel Beauchamp, Saafan Malik, Ann Scher, Sidney R. Hinds

**Affiliations:** ^1^Defense and Veterans Brain Injury Center, Silver Spring, MD, United States; ^2^Henry M. Jackson Foundation for the Advancement of Military Medicine, Bethesda, MD, United States; ^3^Naval Hospital Camp Pendleton, Camp Pendleton, CA, United States; ^4^General Dynamics Information Technology, Falls Church, VA, United States; ^5^Fort Hood Intrepid Spirit Center, Fort Hood, TX, United States; ^6^Fort Bliss Intrepid Spirit Center, Fort Bliss, TX, United States; ^7^Uniformed Services University, Bethesda, MD, United States

**Keywords:** service member, military, concussion, mild traumatic brain injury, headache

## Abstract

**Background:** Headache is a common symptom reported following concussion/mild traumatic brain injury. The Department of Defense's clinical recommendation (CR) describes guidance for primary care providers for the management of post-traumatic headache (PTH) in Service members.

**Objective:** The objective of this study is to examine the association between training on the CR with provider clinical practice, patient behaviors, and symptom recovery.

**Methods:** Participants were healthcare providers and two patient groups (one receiving care as usual [CAU] and another receiving care after provider training on PTH CR [CR+]). Providers were interviewed at three time points: (1) prior to CAU enrollment; (2) after CAU enrollment, but prior to training; and (3) after CR+ follow-up. Data from the second and third provider interview were used to evaluate a potential difference between provider practices pre- and post-training (*n* = 13). Patients were enrolled within 6 months of concussion. Patient outcomes (including neurobehavioral and headache symptoms) were assessed at three time-points: within 72 h (*n* = 35), at 1-week (*n* = 34) and at 1-month post-enrollment (*n* = 27).

**Results:** Most follow-up care reported by providers were recommended within 72 h of initial visit post-training vs. >1 week pre-training. Additionally, providers reported a greater number of visits based on patient symptoms after training than before. Post-training, most providers reported referring patients to higher level of care “as needed,” if not “very rarely,” compared to 25% reported referrals prior to training. At 1-week post-enrollment the CR+ patient group reported more frequent medical provider visits compared to the CAU group. This trend was reversed at the 1-month follow-up whereby more CAU reported seeing a medical provider compared to CR+. By 1-week post-enrollment, fewer patients in the CR+ group reported being referred to any other providers or specialists compared to the CAU group. No differences in patient outcomes by provider training was found.

**Conclusion:** The study results demonstrate the feasibility of training on the Management of Headache Following Concussion CR in order to change provider practices by promoting timely care, and promoting patient compliance as shown through improvement in follow-up visits and more monitoring within the primary care clinic.

## Introduction

Post-traumatic headache (PTH) is a common symptom of mild traumatic brain injury (mTBI) (1–5), with a reported cumulative incidence of 91% for new onset or worsening headaches over 1 year post-concussion in one prospective study, and persistent headache reported for over a third of the study sample (4). Although PTH usually improves in most instances within 6–12 months post-injury, 18–33% may continue to persist well-beyond a year (5). With over 340,000 Service members (SMs) diagnosed with concussion since 2000 (6), mTBI and associated PTH have a tremendous impact on warfighter readiness. A study of veterans from Operation Enduring Freedom/Operation Iraqi Freedom reported 74% with PTH within 30 days of concussion (7). Although full recovery from concussion for SMs typically occurs within 3 months, one study has shown over 45% of this population to have persistent post-concussive symptoms, including PTH, past this period (8). Given the high prevalence of post-concussive PTH in the military, proper management is necessary to facilitate return to duty (9).

Primary care providers (PCPs) are a key component of the management of concussion and its symptoms, along with other pre-existing, comorbid conditions, and doing so during short clinical visits. Given the challenges of PCPs in provider care to patients with PTH such as patient compliance, comorbidities and varying patient presentation, provider level of expertise and complying with mission requirements, many important components of treating PTH such as evaluation for red flags (e.g., Glasgow Coma Scale Score <15, loss of consciousness > 5 min, repeated vomiting, presence of systemic symptoms) and headache history, diagnosis of specific headache type, and utilization of both pharmacological and non-pharmacological approaches (10–12) may be inadvertently overlooked. Further, inadequate follow-up, quick or late patient referrals to higher levels of care, and limited patient education may negatively affect clinical care and, in turn, patient outcome. Standardized care aligned to best practices requires clear standard of care protocols that will help ensure provider familiarity with current clinical recommendations (CR) to treat post-concussive headache, training on those protocols, and aid in complete and proper implementation.

The Management of Headache Following Concussion CR is a Defense and Veterans Brain Injury Center (DVBIC)-led Department of Defense (DoD) CR for primary care on the management of PTH for SMs in deployed and non-deployed military settings (10–12). There are no FDA treatments specifically for PTH and treatment recommendations are based on the primary headache phenotype the PTH most resembles (11, 13). Prior studies have suggested that the PTH phenotype often resembles migraine (14) and may be superimposed on a continuous or constant headache (15–17). Based on published literature and knowledge from subject-matter experts, the CR indicates the importance of performing focused headache history and assessment, followed by the identification and diagnosis of headache type (treating accordingly using both pharmacologic and non-pharmacologic approaches), and identifying concussion or headache red flags (e.g., declining neurological status, Glasgow Coma Scale Score <15, thunderclap headache) (11). The CR also emphasizes the importance of follow-up care, particularly post-deployment, and could potentially improve confidence in treating PTH patients with clear guidelines and, thus, reduce the number of potentially unnecessary referrals to higher level of care (e.g., neurology). This CR was created at the request of providers in the US Armed Services as a means to standardize the management of PTH; however, it is uncertain whether provider knowledge of such guidance (as the CR is readily available) without training is sufficient to change clinical practice (18). Training on the PTH CR may be necessary not only to ensure proper implementation of such guidelines, but also to support dissemination. The implementation of knowledge obtained from CRs into practice is complicated in military medical settings as uniformed military medical providers move regularly as assignments may change with changing operational requirements. This would likely interrupt adequate training and education for all providers who treat PTH in SMs. Beyond proper education of PCPs, it is also important to determine whether such training is translated into improvement in patient behavior to support symptom recovery, which may lead to better patient outcomes. Accordingly, the two primary aims of this study were: (1) to examine the impact of training in the Management of Headache Following Concussion CR on provider clinical practice in the treatment of PTH; and (2) to demonstrate the potential benefit of the provider training on the PTH CR on patient behavior and outcomes during symptom recovery.

## Materials and Methods

**Figure 1:**
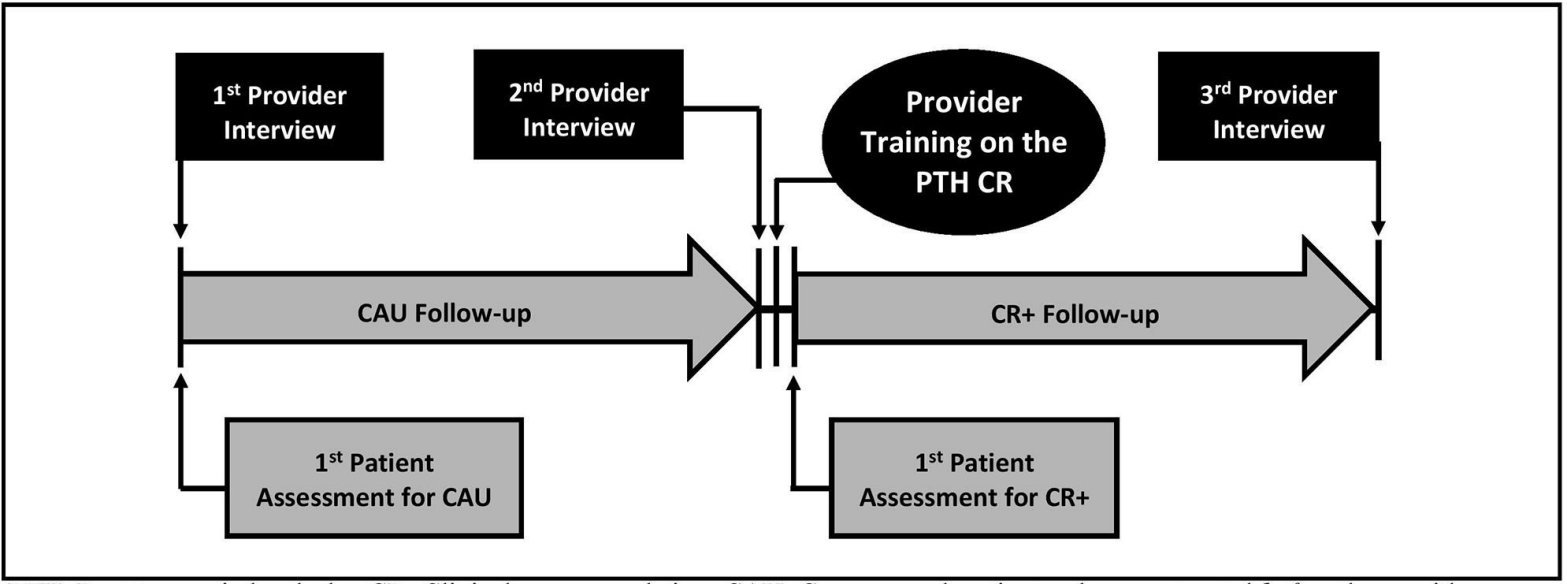
Diagram of study procedure for provider and patient data collection and provider training. PTH, Post-traumatic headache; CR, Clinical recommendation; CAU, Care as usual, patients who received treatment *before* provider training on the clinical recommendation; CR+, Patients who received treatment *after* provider training on the clinical recommendation.

Data were obtained from research evaluating the potential benefit of the Management of Headache Following Concussion Clinical Recommendation, and consisted of both provider and patient data. There were two patient groups: one who received care as usual (CAU group) prior to providers receiving training on the CR and one who received care after providers received training on the CR (CR+ group). The group to which patients were assigned depended on the period when they were enrolled in the study (i.e., prior to or after provider training). Training on the CR was done in groups and multiple sessions were available to meet providers' schedules. There were 35 patients enrolled within 6 months of concussion. And interviewed within 72 h post-enrollment (CAU *n* = 21, CR+ *n* = 14), at 1-week follow-up (CAU *n* = 20, CR+ *n* = 14), and at 1-month follow-up (CAU *n* = 15, CR+ *n* = 12). Providers were interviewed at three time points: (1) prior to the enrollment of the CAU group; (2) after the CAU group completed follow-up, but before receiving the CR+ training; and (3) after the CR+ group completed follow-up (see Figure 1). For the purpose of this study, the focus will be on data from the second and third provider interview to evaluate a potential difference between provider practices before and after the CR training. A total of 13 providers were observed before and after CR training.

### Study Participants

The participants for this study were identified and recruited from two Army military training facilities in the southwest where concussed SMs sought care for their clinical management. The provider participants were medical staff members (i.e., physicians, physician assistants, and nurse practitioners) who worked at one of these two U.S. Army military treatment facilities and provided care for concussed SMs. The study was made known to providers through face-to-face encounters with a study investigator, regular clinic meetings where study announcements were made informing or reminding attendees about local military treatment facility research opportunities and research staff points of contact, and/or approved research flyers posted in clinics. Patient participants had seen an enrolled provider, had received a concussion diagnosis within the past 6 months and reported headaches beginning or worsening after the concussion. Patients were enrolled within 72 h of their initial visit. Consistent with other studies (4, 9, 19), the International Classification of Headache Disorders requirement for headache onset or worsening did not have to occur within 7 days of injury (20).

### Measures and Procedures

Data from provider participants was qualitative in nature, derived from semi-structured interviews with the provider participant and researcher. Patient data was obtained using medical record review as well as self-report questionnaires which include evaluation of headache history, neurobehavioral, and headache symptoms and lifestyle administered face-to-face by a civilian research staff member. During consent, the research staff described the study protocol, specifying that participation was voluntary, not mandatory, and not command-directed, and conducted the provider or patient assessment soon after. The study was reviewed and approved by the Brooke Army Medical Center Human Research Protections Office. The protocols used in the study complied with all applicable regulations through implementation of the Brooke Army Medical Center Human Research Protection Program and administration of the Regional Health Command-Central Institutional Review Board.

#### Provider Interview Structure, Content, and Implementation

Each provider semi-structured interview was conducted in-person by research staff (on a one-on-one basis), in a dedicated space (e.g., exam room, clinician office). The use of standardized questionnaires with both open- and close-ended questions, along with training interviewers to strictly adhere to the question and answer format questions, was conducted to avoid introducing interviewer bias. A Phillips DVT 6000 recorder was used to record interviews which were subsequently transcribed by one research staff member and reviewed and verified by another. All provider participants received the same questions, with follow-up questions permitted as needed to allow for elaboration of participant responses. Multiple responses were allowed to these questions. Transcribed responses were assigned to predetermined codes that were theoretically- or empirically-based. Each transcript was coded by two study investigators and discrepancies were discussed by raters to reach consensus. Remaining discrepancies were resolved through discussion among the research group until consensus was reached. Further refining of coded responses was done to further collapse the number of categories by combining similar response categories into newly-coded variables (e.g., patient-related factors). Newly coded variables were created based on consensus from separate members of the research team with expertise in psychology and epidemiology. Supplementary Table 1 lists the questions from the semi-structured second and third provider interviews.

#### Patient Assessment

Patient data included demographics, headache history, follow-up and referral, lifestyle, neurobehavioral symptoms, and headache-specific symptom. *Demographic characteristics* were obtained at the time of enrollment and included age, sex, and education. *Headache history* was assessed at the time of enrollment and included questions that evaluated the timing of headaches and their characteristics at its worse (e.g., painfulness, location, and visual changes). To determine the frequency of *follow-up* and the number of *referrals* to other providers or specialists, patients were asked “How many times have you seen a medical provider for your head injury? If you have completed this questionnaire before, how many times have you seen a medical provider since the last time you completed this questionnaire?” and “Have you been referred to any other providers or specialists?,” respectively. Information on patient *lifestyle* was obtained by asking questions regarding the average number of the following in the last 2 weeks: (1) cups of caffeinated drinks consumed per day, (2) h of sleep per night, (3) cigarettes smoked per day, (4) alcoholic beverages consumed per day, (5) meals consumed per day, and (6) glasses of water consumed per day. *Neurobehavioral symptoms* were evaluated using the Neurobehavioral Symptom Inventory (21) which has previously demonstrated high internal consistency among SMs (total alpha = 0.95; subscale alpha = 0.88–0.92) (16). The Neurobehavioral Symptom Inventory was administered during enrollment and at all follow-up periods. The 22-items were grouped into the following categories based on previous exploratory factor analyses: cognitive, vestibular, somatosensory, and affective symptoms (22, 23). For each item, responses included “none” (0), “mild” (1), “moderate” (2), “severe” (3), and “very severe” (4). Additionally, the Neurobehavioral Symptom Inventory item specific for *headache symptom* was evaluated individually.

### Statistical Analyses

Descriptive statistics were presented as medians (interquartile range [IQR]) for continuous variables and as frequencies (percentages) for categorical variables. Provider data was assessed by interview time (i.e., second vs. third) as this differentiates between pre- and post-training, respectively. Patient data was evaluated based on the cohort in which the providers treated before and after training (CAU vs. CR+). Differences in medians were analyzed using Wilcoxon-signed rank sum test (for provider data to take into account within person change) and Wilcoxon-rank sum test (for patient data). Differences in frequencies were evaluated using Chi-square tests. Pairwise deletions were used in analyses to handle missing data. Where applicable, patient data was evaluated at each time point (i.e., at the time of enrollment, 1 week post-enrollment and 1 month post-enrollment), as well as the change from study enrollment to 1 week and 1 month post-enrollment, specifically for change in the frequency of patient-reported follow-up). The primary analysis focused on change in the frequency of patient-reported follow-up from the time of study enrollment to 1 week post-enrollment, as this was around the period in which most recoveries have been reported (24–28).

Significant *p*-value was considered at a level of < 0.05. As results from this study were preliminary, findings should be interpreted as exploratory. All analyses were completed using Stata statistical software (Stata, RRID:SCR_012763), release 15 (StataCorp, 2017, College Station, TX).

## Results

### Provider Report (*n* = 13)

#### Background

The provider population is described in Table 1. There was a relatively equal number of civilian and military providers in the study, most of whom were Physician's Assistants. There was a median of 9 years practicing medicine (IQR = 2.5, 20, range = 0.5–25), and a median of 7 years treating patients with post-concussive headaches (IQR = 2.5, 15, range = 0.5–17). Toward the end of the study, providers treated a median of 4 PTH patients (IQR = 2, 8) per month (range = 0–28).

**Table 1:** Provider characteristics (*n* = 13).

**Provider results**		
	**Estimate**	**Range**
**Site**, ***n*** **(%)**		
Fort Hood	9 (69.2)	NA
Fort Bliss	4 (30.8)	NA
**Civilian or military**, ***n*** **(%)**		
Civilian	6 (46.2)	NA
Military	7 (53.9)	NA
**As a healthcare provider, what is your professional role?** ***n*** **(%)**		
Physician (DO, MD)	3 (23.1)	NA
Physician Assistant	8 (61.5)	NA
Nurse Practitioner	2 (15.4)	NA
**For how many years have you been practicing? Median (IQR)**	9 (2.5, 20)	0.5–25
**For how many years have you been treating patients with headache following concussion? Median (IQR)**	7 (2.5, 15)	0.5–17

#### Familiarity With the CR

Prior to the training (during the second interview), 76.9% (*n* = 10) of providers reported familiarity with DVBIC's CR for the Management of Headache Following Concussion. This percentage increased after receiving training (92.3%, *n* = 12); however, not Statistically different from prior to training. It is important to note that during the first interview, providers were asked about their training, education and comfort with treating PTH. Although the CR was not mentioned in the first interview, individuals might have sought out information regarding the CR because the research was being conducted by DVBIC. This might have contributed to the percentage of providers who reported familiarity with DVBIC's CR, and, as such, the percentage might be an overestimation of actual current clinical practice prior to CR training.

Providers were asked to describe the key principals of the CR as presented in Table 2. The key principles of the CR reported included pharmacological treatment (23.1%), patient education (11.5%) and specialist referral (11.5%), as well as no activity (defined as no physical activity, quarters and no visual or cognitive stimulation) (Table 2).

**Table 2:** Provider-reported key principles of the Management of Headache Following Concussion Clinical Recommendation (CR) post-training (*n* = 13).

**Provider results**		
**Question item**	**Response**	**Frequency (%)**
**Please briefly describe the key principles in the Management of Headache Following Concussion CR**	Concussion profile	2 (7.7)
	Avoid offending activity	1 (3.8)
	Patient education	3 (11.5)
	CR talks about pharmacological treatment	6 (23.1)
	Specialist/higher level of care referral	3 (11.5)
	Concussion red flag^a^	2 (7.7)
	No activity^b^	5 (19.2)
	Ambiguous rest^c^	1 (3.8)
	Headache management	1 (3.8)
	Military acute concussion evaluation (MACE)	1 (3.8)
	Limit caffeine	1 (3.8)
	**TOTAL**	**26 (100.0)**

Providers were allowed multiple responses.

a *Concussion red flag includes progressively declining level of consciousness (LOC), LOC > 5 min, declining neurological status, Glasgow Coma Scale Score <15, seizures, neurological deficit (motor or sensory), cannot recognize people or disoriented to place, repeated vomiting, worsening headache, pupil asymmetry, double vision, slurred speech, and unusual behavior*.

b *No activity is defined as no physical activity, quarters and no visual or cognitive stimulation*.

c *Ambiguous rest is defined as general activity or non-specific when providers indicated “rest” but did not specify bed rest, nor indicated activity should be limited*.

#### Challenges in Care and Differences in Care Provided

Prior to the training, providers rated a median of 8 (out of 10, with 10 achieving the greatest level) in their comfort providing care to the patients with post-concussive headaches (IQR = 7, 9.8, range = 5–10). This median increased to 10 (IQR = 9, 10, range = 8–10) after receiving the training which was significantly higher than prior to training (*p* = 0.008). Interestingly, the challenges reported in provider care for patients with post-concussive headaches prior to training included military-related factors (i.e., command, military in general, mission requirements) that were no longer reported after the training (Table 3). On a scale of 1 to 10, with 10 being the most similar, providers reported a median scale of 8 (IQR = 8, 9.3, range = 5–10) as to the similarity of approach they provided from one headache patient to the next prior to receiving training. This was significantly higher than the post-training report of 7 (IQR = 5, 8, range = 1–9, *p* = 0.020), albeit by only 1 unit. Further, many of the factors reported to contribute to differences in care prior to training (e.g., symptomatology, patient-related factors [age, patient history, behavioral health history, physical exam], injury-related factors [TBI history, time from injury onset, location, neurological findings, characteristic of headache] and mechanism of injury) were not reported post-training (Table 3).

**Table 3:** Provider-reported challenges in providing care and differences in care provided for patients with post-concussive patients, pre- and post-training (*n* = 13).

**Provider results**			
**Question item**	**Responses**	**Frequency (%)**	
	**Broad categories**	**Pre-training**	**Post-training**
**Can you describe some of the challenges in providing care for patients with headache following concussion?**	None	1 (4.2)	1 (5.3)
	Patient-related factors^a^	12 (50.0)	13 (68.4)
	Provider-related factors^b^	6 (25.0)	5 (26.3)
	Military-related factors^c^	4 (16.7)	0
	Co-morbidities	1 (4.2)	0
	**TOTAL**	**24 (100.0)**	**19 (100.0)**
**What factors contribute to differences in care you provide from one patient to the next?**	Medication	5 (17.2)	1 (10.0)
	Comorbidities	2 (6.9)	1 (10.0)
	Injury-related factors^d^	10 (34.5)	5 (50.0)
	Symptomatology	2 (6.9)	0
	Patient-related factors^e^	10 (34.5)	3 (30.0)
	**TOTAL**	**29 (100.0)**	**10 (100.0)**

a Examples of patient-related factors include follow-up, compliance, expectations, history, fear of stigma, and secondary gain.

b *Examples of provider-related factors include varying treatment plans, and inability to enforce treatment care*.

c *Examples of military-related factors include command, and military in general*.

d *Examples of injury-related factor include severity of injury, types of headaches, time from onset of injury, neurological findings, and TBI history*.

e *Examples of patient-related factors include compliance, age, patient history, behavioral health history, and visual changes*.

#### Recommendation of Follow-Up and Referral to Higher Level Care

Although the number of follow-up care visit recommendations remained relatively unchanged between pre- and post-training (84.6%, *n* = 11 vs. 100.0%, *n* = 13, respectively), the occurrence differed in that, after provider training, most follow-up care was recommended within 72 h post-enrollment (63.7%), which was earlier compared to more than 1 week as recommended by providers prior to the CR training (50%) (see Table 4). This, however, was not statistically different (*p* = 0.280). Additionally, determination of the number of visits based on patient symptoms, promoting individualized care, were reported more after the training than before (69.2 vs. 53.9%, respectively, Table 4). Of note prior to the enrollment of CAU patients, majority of providers recommended 2 or more clinical visits, as well as recommendations of visits within 72 h post-enrollment which was analogous to post-training timing of follow-up.

**Table 4:** Provider recommendation of follow-up and referral to higher level of care (*n* = 13).

**Provider results**		
**Question items**	**Frequency (%)**	
	**Pre-training**	**Post-training**
**Do you typically recommend any medical follow-up to patients?**		
Yes	11 (84.6)	13 (100.0)
No	0	0
It depends	2 (15.4)	0
**When do you recommend the visits occur (1 day later or 1 week)?** ***n*** **=** **11, 12**		
≤24 h	0	1 (9.1)
24–48 h	3 (24.9)	2 (18.2)
48–72 h	2 (16.7)	4 (36.4)
1 week	1 (8.3)	2 (18.2)
>1 week	6 (50.0)	2 (18.2)
**How many follow-up visits with the patient do you recommend?**		
0 visits	0	1 (7.7)
1 visit	3 (23.1)	0
2 or more visits	3 (23.1)	2 (15.4)
Depends on symptoms	7 (53.9)	9 (69.2)
**How often do you refer patients to rehabilitation provider and/or higher level of care?** ***n*** **=** **12**		
Very rarely	NA	3 (25.0)
Occasionally	NA	2 (16.7)
Often	NA	2 (16.7)
As needed	NA	5 (41.7)

Prior to training, providers reported 75% of their patients follow the recommendations provided (IQR = 50, 82.5, range = 0–100). This was significantly higher than the reported 50% after training (IQR = 50, 70, range = 35–100, *p* = 0.002).

Prior to training (during the second interview), providers reported referring a median of 25% (IQR = 10, 67.5, range = 5–100) of their patients with headache to a rehabilitation provider and/or higher level of care. It is important to note that during the first provider interview, there were 50% of patients who were referred to higher level of care by providers in the study (29). As previously mentioned, the CR was not mentioned in the first interview; however, providers might have sought out information regarding the CR because the research was being conducted by DVBIC. This might, in turn, contribute to an underestimation by the second interview. After the training, providers reported referring patients to rehabilitation provider and/or higher level of care as needed, if not, “very rarely.” The type of specialist and the factors that determine referral was similar pre- and post-training; the most common referral was to neurologists (50.0 and 36.8%, respectively) and TBI (45.0 and 42.1%), and the most common factor that determined a referral to be made was symptomatology.

#### Education

Prior to training, 45.5% of providers (*n* = 5) reported distributing the CR's patient education materials. Though not shown statistically different (*p* = 0.441), this percentage increased to 61.5% (*n* = 8) post-training. Verbal instruction was also reported by 81.8% (*n* = 9) of providers prior to training, which increased to 100% (*n* = 12) post-training.

### Patient Report (*n* = 35)

#### Patient Background

Table 5 describes the patient population. The patient participants had a mean age of 24.1 years (*SD* = 4.5), were mostly men (80%, *n* = 28) and never married. About half had some college (1–3 years or technical school) and most were either junior enlisted (E1–E3, 48.6%) or non-commissioned officers (E4–E6, 45.7%). The median time from injury to the first patient assessment was 21 days (IQR = 8, 39). None of these characteristics differed between the CAU vs. the CR+ group.

**Table 5:** Patient characteristics, overall and by intervention status (*n* = 35).

**Patient results**				
**Variables**	**Overall**	**Intervention status**		
		**CAU (*****n*** **=** **21)**	**CR+** **(*****n*** **=** **14)**	***p***
**Age in years**, ***mean*** **(*****SD*****)**	24.1 (4.5)	24.3 (5.1)	23.6 (3.7)	0.665
**Sex**, ***n*** **(%)**				0.490
Men	28 (80.0)	16 (76.2)	12 (85.7)	
Women	7 (20.0)	5 (23.8)	2 (14.3)	
**Education**, ***n*** **(%)**				0.214
High school diploma or GED	17 (48.6)	12 (57.1)	5 (35.7)	
Some college (1–3 years or technical school)	18 (51.4)	9 (42.9)	9 (64.3)	
**Marital status**, ***n*** **(%)**				0.919
Married	13 (37.1)	7 (33.3)	6 (42.9)	
Never married	17 (48.6)	11 (52.4)	6 (42.9)	
Divorced	2 (5.7)	1 (4.8)	1 (7.1)	
Other	3 (8.6)	2 (9.5)	1 (7.1)	
**Rank**, ***n*** **(%)**				0.511
Junior enlisted (E1–E3)	17 (48.6)	11 (52.4)	6 (42.9)	
Non-commissioned Officers (E4–E6)	16 (45.7)	8 (38.1)	8 (57.1)	
Senior non-commissioned officers (E7–E8)	2 (5.7)	2 (9.5)	0 (0.0)	
**Days since injury**, ***median (IQR)***	21 (8, 39)	21 (8, 38)	22 (13, 44)	0.522

*CAU, care as usual patient group; CR+, clinical recommendation patient group; SD, standard deviation; IQR, interquartile range*.

#### Headache History

In the present study, 94.3% reported headache onset or worsening within 7 days of injury, while 5.7% reported delayed-onset or worsening (i.e., more than 1 week post-injury). All patients reported having headaches every day, either in the form of continuous headaches (51.4%), or daily, but not continuous headaches (48.6%). On a scale of 1 to 10, with 10 as the highest intensity, patients reported a median of 8 (IQR = 7, 10) with regards to headache pain at its worse which occurs on both sides for 60% of the participants; 85.7% stating that the pain is confined to a particular location. The headache pain characteristics included throbbing or pulsating (for 91.4%) and worsening with physical activity (80.0%). Additionally, patients stated being bothered by light (80.0%) and noise (68.6%) more than usual during the headache, as well as feeling sick to their stomach or even vomiting (45.7%). Most patients reported seeing spots, stars, flashing lights, zig zag lines, or loss of vision with their headaches (74.3%) and this was reported significantly more by the CR+ group compared to the CAU group (92.9 vs. 61.9%, *p* = 0.040) (see Supplementary Table 2).

#### Patient Follow-Up and Referral to Higher Level of Care

The CR+ group reported more frequent visits to a medical provider for their head injury compared to the CAU group at 1-week post-enrollment (median = 1.5, IQR = 0, 2 vs. median = 0, IQR = 0, 2, respectively, *p* = 0.014, Table 6). When change in the frequency of patient-reported follow-up was evaluated from study enrollment to 1-week post-enrollment, no significant difference was found (Table 6). The reverse pattern was seen at the 1-month follow-up whereby more CAU patients saw a medical provider for their head injury compared to the CR+ group (median = 1.5, IQR = 1, 3 vs. median = 0, IQR = 0, 1, respectively, *p* = 0.009, Table 6). When change in the frequency of patient-reported follow-up was evaluated from study enrollment to 1 month post-enrollment, the results were null (Table 6). Additionally, by 1-week post-enrollment, fewer CR patients than CAU patients reported being referred to any other providers or specialists (*n* = 14, 70.0% vs. *n* = 5, 35.7%, respectively, *p* = 0.048, Table 6).

**Table 6:** Patient-reported clinical care received (follow-up, referral and education)—median (IQR) / frequency (%), by intervention status.

**Patient results**			
	**Intervention status**		
	**CAU (*****n*** **=** **21)**	**CR+** **(*****n*** **=** **14)**	***p***
**How many times have you seen a medical provider for your head injury (or last time you completed this questionnaire)?** ***Median (IQR)***			
T0 (≤72 h) (*n* = 34)	3 (2, 5)	3 (2, 4)	0.530
T1 (1 week) (*n* = 33)	0 (0, 1)	1.5 (0, 2)	0.014^*^
T2 (1 month) (*n* = 25)	1.5 (1, 3)	0 (0, 1)	0.009^*^
Change from T0 to T1	−3 (−5,−1), *n* = 18	−2 (−3, 0), *n* = 14	0.073
Change from T0 to T2	−2 (−3, 0), *n* = 14	−3 (−4,−1), *n* = 11	0.328
**Have you been referred to any other providers or specialist? Yes**, ***n*** **(%)**			
T0 (≤72 h) (*n* = 35)	10 (47.6)	3 (21.4)	0.116
T1 (1 week) (*n* = 34)	14 (70.0)	5 (35.7)	0.048^*^
T2 (1 month) (*n* = 28)	5 (33.3)	5 (41.7)	0.656
**Did you receive from your provider any education materials related to managing a concussion? Yes**, ***n*** **(%)**			
T0 (≤72 h) (*n* = 35)	8 (38.1)	8 (57.1)	0.435
T1 (1 week) (*n* = 34)	7 (35.0)	9 (64.3)	0.092
T2 (1 month) (*n* = 24)	4 (33.3)	7 (58.3)	0.219

CAU, care as usual patient group; CR+, clinical recommendation patient group; IQR, interquartile range.

* *Significant at a p < 0.05*.

#### Patient Assessment

Among lifestyle changes, the patients treated after providers received training drank 2 more glasses of water per day (IQR = −2, 7) within 1 week of enrollment compared to patients treated before the providers received training who had no change (IQR = −1.8, 0, *p* = 0.040). No other significant lifestyle changes were found (Supplementary Table 3). There was also no statistical difference between the CR+ and CAU group with regards to neurobehavioral and headache symptoms at any timepoint or change from around the time of enrollment to either 1 week or 1 month post-enrollment (see Supplementary Table 4).

#### Education

At all time points, there was a greater percentage of CR+ patients who received education materials related to managing a concussion from their providers compared to CAU patients; however, only at 1 week post-enrollment was the difference marginally significant (*p* = 0.092).

## Discussion

This study benefited from the use of both patient and provider data at multiple time points. Overall, our preliminary results suggest a benefit of provider training in the CR on clinical care for the management of headache following concussion. First, provider training may be contributing to clinical practices in encouraging follow-up visits of patients in a timely manner. CR+ patients reported greater number of provider visits than CAU patients within 1 week of study enrollment, which was consistent with reports from most providers reporting to recommended earlier follow-up (i.e., most within 72 h post-enrollment). Although CAU patients had a greater number of visits by 1 month compared to CR+ patients, this may suggest that CR+ patients may have recovered more efficiently by following up earlier compared to CAU patients who may have more visits at a later period due to symptomatology that have not been addressed and persisted, or even worsened, calling for the need for a provider visit. Thus, provider visits among the CAU group might have been driven by inefficient follow-up care that was otherwise endorsed by provider training. Although headache and neurobehavioral symptoms were not significantly different between patient groups, other ailments not specific to these evaluations (e.g., post-traumatic stress disorder) may be being addressed by guidelines of the CR that may contribute to later improvement not assessed in this study. Second, the significantly fewer referrals to higher levels of care for the CR+ group at 1 week post-enrollment may suggest that training may increase comfort and knowledge among providers in treating PTH patients at the primary care level. This was supported by provider data in which comfort level in providing care for PTH patients increased after training. Further, some reported challenges in care and factors that contribute to differences in care (e.g., patient- and military-related factors) were no longer reported after training, which may have endorsed greater frequency of follow-up in a timely manner, as well as reserving referrals based on patient need.

Follow-up care has been shown associated with positive health outcomes (30, 31). Done frequently and in a timely manner, this may potentially shorten recovery time (31) as patient symptoms, along with progression of treatment response, are more closely monitored to determine if changes in treatment are necessary. As previously mentioned, our study found CR+ patients to more frequently engage in follow-up for their head injury and at an earlier time (i.e., within 1 week post-enrollment vs. 1 month) compared to CAU patients. This may signify more prompt and accurate treatment for CR+ patients and, thus, less likely for later follow-up as seen among CAU patients. This may, in turn, require less referral to higher level of care if symptoms are addressed early enough to prevent or slow progression or exacerbation. Addressing symptoms at lower levels of care offers the added effect that the CR could achieve the same or near same level of clinical outcome that a specialist could attain (e.g., time to resolution, level of pain reduction, number of visits, etc.) if symptoms were allowed to persist. Also, while these differences did not translate to differences in patient outcomes assessed in the study between CR+ and CAU patients, studies with a larger sample size may be needed for more accurate assessment of significance, along with an expansion of the outcomes to include other measures that may have been more directly affected by more efficient follow-up and less referrals.

It has been suggested that proper initial education distributed early during recovery has a positive impact on post-concussive symptoms (32). Although we found a non-significant increase in reported education distribution by providers from pre- to post-training, timing of dissemination could not be evaluated (i.e., providers were generally asked about their dissemination of education after all patients completed follow-up but not timing of distribution). Early distribution of education may be more effective on both patient compliance and outcome, and more frequent follow-up may reinforce the content of educational materials. The combination of early education and follow-up may thus lead to better concussion recovery. A larger sample would also be needed for such an assessment.

Contrary to what was expected, there was less percentage of CR+ vs. CAU patients who followed the CR as reported by providers. This may perhaps reflect the need to better train PCPs on communicating such recommendations to patients. Future studies might need to identify and address barriers for providers to successfully communicate the PTH CR so that patients have a thorough understanding of what are expected of them. Studies that investigate the reasons why PCPs do not provide the CR (e.g., did not like the CR, simply forgot, difficulty in accessing, etc.) might additionally be of benefit. It is important to note that the study did not assess what particular provider recommendation was not followed; hence, it could not be determined whether such recommendation aligned with best clinical practice. For example, a few providers reported “no activity” as a key principle of the PTH CR. This may or may not be in line with the CR depending on interpretation. Some patients may be increasing their activity level as symptoms subside, which is congruent to the guidelines. As such, along with successful communication of the PTH CR, it is also important that providers are completely well-versed on the details of the guidelines and themselves adhere to such recommendations, but still consider individual needs. Conversely, the lower percentage of reported patients who followed the CR after training vs. before might be due to increased provider awareness of the CR after training, leading to more rigorous assessment in patient compliance with the CR. Further, with such rigorous assessment and/or a stricter guidance by providers, this might pose as a greater challenge to patients, suggesting greater support might be needed (perhaps done during more frequent and earlier follow-up to better monitor patient compliance) for patients to adhere to the CR.

Timely referrals to higher level of care is necessary to address concerning symptoms that require more rigorous treatment to remedy. However, unnecessary referrals may pose an overburden on these resources when less urgent care may be tackled at primary care facilities. Quick referral may be indicative of provider lack of knowledge or comfort in treating PTH cases that require greater attention, but possibly not at the level of higher rehabilitative care, and can otherwise be addressed in primary care settings. Our findings using patient response suggests that some of these obstacles may have been addressed with provider training on the CR, whereby greater referrals were reported during pre-training during the early stages post-enrollment (i.e., within 1 week) compared to post provider training. Further, after training on the CR, most providers reported referring patients to higher level of care “as needed,” if not “very rarely.” This suggests that individualized care is being recognized by most of the study providers as being an important aspect to treating PTH and that this may be contributing to less referrals to higher level care. Further, the greater comfort level in treating PTH patients after training than before may help sustain such individualized care as more providers grow more confident in their skills and knowledge to treat PTH.

Since clinical visits are relatively short, it is important that providers not only receive proper education on the PTH CR, but are also trained in efficiently implementing the guidelines that meets this time constraint, while not sacrificing important aspects of the CR. Some of this training may already be addressed by the current PTH CR as shown with increased comfort in treating PTH patients and reduction in challenges reported by providers in providing care, particularly military-related factors (e.g., mission requirements). A greater frequency and earlier follow-up as consistently reported by both provider and patients also support improvement after training in the CR.

It is important for practitioners to have the skills and clinically relevant tools to optimize accurate symptom management. The benefits of early and accurate TBI diagnosis and systematic follow-up regarding symptoms, such as PTH, are well-documented and have been the focus of several longitudinal studies (8, 33, 34). Inadequate or inaccurate treatment of PTH may result in transformation to chronic daily headache or the additional management challenge of medication-triggered rebound headache (35). Persistent headache can incite or worsen mood disorders, insomnia, and cognitive impairment, all of which can affect functional outcome (35–37). Proper and timely management of PTH is essential to avoid further worsening of symptoms (5), which not only affect functional outcomes of the individual SM but may also impact unit readiness and, consequently, force readiness. Sub-optimal force readiness places both the injured SM and members of his or her unit at risk. Timely and accurate intervention following concussion promotes expedited recovery of concussion symptoms (31) and enables SMs a more timely and safe return to duty.

There were several limitations in our study to note. For example, certain pertinent details to treating PTH such as headache type were not asked of providers to more accurately depict whether providers follow the DoD CR. However, this more general interview allowed for more provider-led responses. As previously mentioned, although providers reported patient non-compliance with recommendations, it would have been informative to know what recommendations were not followed to determine whether such suggestions aligned with the PTH CR. Further, we did not evaluate the context of what providers meant by “no activity” as a key principle of the CR. This response may have been specific for patients whose headache symptoms may be severe or remedied by “no activity,” reflecting individualized treatment, which is aligned with best clinical practice. Studies that provide a more granular assessment of these questions may provide a more accurate depiction of both provider and patient compliance with the PTH CR. Although the semi-structured provider interviews did not assess whether providers altered their treatment based on patient history (e.g., comorbidities, family history of headache, medication), for the purpose of this study, the authors felt this line of inquiry was outside the scope of this study. This study was also limited to a population of active duty SMs, mostly males, and, thus, would be generalizable only to such population. However, our findings still hold importance as this is a high-risk group for concussion due to their occupational demands, which makes prompt recovery essential, not only to avoid compiling injury from successive concussions and complicating recovery, but also to return to duty and ensure warfighter readiness. Information on pre-existing conditions (e.g., migraine, other primary headache disorders, psychological conditions) that may attribute to persistent PTH development (38) was also not available. Additionally, we had a small provider and patient sample size which may not have been conducive to detecting significance in some of our analyses. Reassessment of our results in a larger sample size may be valuable in validating the findings of this study. Lastly, the qualitative data produced from this study were grouped subjectively based on common themes and quantified accordingly. However, to minimize miscategorization, these groupings were conducted by a team with expertise in medicine, epidemiology, mTBI/concussions and PTH. Despite these limitations, the study had strengths which included having rich data on both provider and patients that help in determining consistency in reports and a longitudinal design for both groups.

The results presented in this study demonstrate the potential of the Management of Headache Following Concussion CR to promote provider knowledge and confidence in treating PTH in a military setting. This in turn may translate to timely care, promotion of patient compliance as shown through improvement in follow-up visits and more monitoring within the primary care clinic, and, ultimately, timely return to duty.

## Data Availability Statement

Information and the policies regarding limitations on sharing DoD/DHA data publicly, without an approved Data Sharing Agreement Application (DSAA) can be found at the following website (https://www.health.mil/Military-Health-Topics/Privacy-and-Civil-Liberties/Submit-a-Data-Sharing-Application). The specific DoD Directive (DoDD) that speaks to why we cannot simply share data, even a minimal, de-identified dataset, is the documentation titled “DoDD 5400.11”. In order to access DoD/DHA data, a DSAA must be submitted to the Privacy Office. The appropriate point of contact to initiate the DSAA process can be reached at DHA.DataSharing@mail.mil. This DSAA would be between DVBIC and the intended recipient of the data and would need to be requested by the recipient and signatures obtained from all party's authorities. The DSAA would outline the intended use and retention of the data, which will be reviewed by the Privacy Board (estimated review time is between 3–6 months depending on the type of request). A determination will then be made by the Privacy Board based on whether the intended use of the data by the recipient meets the standards of the DoD Privacy Program. Approval of DSAA is subject to Privacy Board review. For further information, questions can be submitted to the Privacy Office at DHA.DataSharing@mail.mil.

## Ethics Statement

The studies involving human participants were reviewed and approved by The Brooke Army Medical Center Human Research Protection Program and administration of the Regional Health Command-Central Institutional Review Board. The patients/participants provided their written informed consent to participate in this study.

## Author Contributions

RR-B, SK, HF, and EG were involved in the initial concept development for this manuscript. SK lead and coordinated data collection. SK, SE, SS, and DB were instrumental in the acquisition of data. AS, SM, and SH were subject-matter experts on posttraumatic headache. SH and DB provided insight into the clinical relevance of findings. RR-B conducted the descriptive and statistical analyses. The interpretation of data as well as the revision of the manuscript for intellectual content was done by RR-B, SK, HF, EG, DB, SM, AS, and SH. All authors participated in the editing and approval of the final manuscript.

## Conflict of Interest

RR-B was employed by Henry M. Jackson Foundation for the Advancement of Military Medicine. SK and HF were employed by General Dynamics Information Technology. The remaining authors declare that the research was conducted in the absence of any commercial or financial relationships that could be construed as a potential conflict of interest.

## Abbreviations

CR: Clinical recommendation
PTH: Post-traumatic headache
CAU: Care as usual. Patients who received treatment before provider training on the clinical recommendation
CR+: Patients who received treatment after provider training on the clinical recommendation
mTBI/TBI: Mild traumatic brain injury / Traumatic brain injury
SMs: Service members
PCPs: Primary care providers
DVBIC: Defense and Veterans Brain Injury Center
DoD: Department of Defense.

## References

[1] Gurr B Coetzer B. The effectiveness of cognitive-behavioral therapy for post-traumatic headaches. Brain Injury. (2005) 19:481–91. 10.1080/02699050400005176 16134736

[2] Lucas S. Headache management in concussion and mild traumatic brain injury. Phys Med Rehabil. (2011) 3:S406–12. 10.1016/j.pmrj.2011.07.016 22035683

[3] Patil V St. Andre J Crisan E Smith B Evans C Steiner M. Prevalence and treatment of headaches in veterans with mild traumatic brain injury. Headache. (2011) 51:1112–21. 10.1111/j.1526-4610.2011.01946.x 21762135

[4] Lucas S Hoffman J Bell K Dikmen S. A prospective study of prevalence and characterization of headache following mild traumatic brain injury. Cephalalgia. (2014) 34:93–102. 10.1177/0333102413499645 23921798

[5] Lew HL Lin PH Fuh JL Wang SJ Clark DJ Walker WC. Characteristics and treatment of headache after traumatic brain injury: a focused review. Am J Phys Med Rehabil. (2006) 85:619–27. 10.1097/01.phm.0000223235.09931.c0 16788394

[6] Defense and Veterans Brain Injury Center DoD worldwide numbers for TBI worldwide totals. DoD TBI Worldwide Numbers Since 2000. (2019) Available online at: http://dvbic.dcoe.mil/dod-worldwide-numbers-tbi (accessed July 25, 2020).

[7] Theeler B Flynn FG Erickson JC Headaches after concussion in US soldiers returning from Iraq or Afghanistan. Headache. (2010) 50:1262–72. 10.1111/j.1526-4610.2010.01700.x 20553333

[8] Schwab K Terrio HP Brenner LA Pazdan RM McMillan HP MacDonald M. Epidemiology and prognosis of mild traumatic brain injury in returning soldiers: a cohort study. Neurology. (2017) 88:1571–9. 10.1212/WNL.0000000000003839 28314862

[9] Metti A Schwab K Finkel A Pazdan R Cole W Terrio H. Post-traumatic vs non-traumatic headaches: a phenotypic analysis in a military population. Neurology. (2019) 94:e1137–49. 10.1212/WNL.0000000000008935 31924681

[10] Tfelt-Hansen P Pascual J Ramadan N Dahlof C D'Amico D Diener H-C. Guidelines for controlled trials of drugs in migraine: 3rd edition. A guide for investigators. Cephalalgia. (2012) 32:6–38. 10.1177/0333102411417901 22384463

[11] Defense and Veterans Brain Injury Center Management of Headache Following Concussion/Mild Traumatic Brain Injury: Guidance for Primary Care Management in Deployed and Non-Deployed Setting. (2016) Available online at: https://pueblo.gpo.gov/DVBIC/pdf/DV-4309.pdf (accessed July 7, 2019).

[12] Hinds SR Livingston SC. Traumatic brain injury clinical recommendations: impact on care and lessons learned. US Army Med Dep J. (2016) (2–16):97–101. 27215874

[13] Theeler B Lucas S Riechers RG II Ruff RL. Post-traumatic headaches in civilians and military personnel: a comparative, clinical review. Headache. (2013) 53:881–900. 10.1111/head.12123 23721236

[14] Lucas S Ahn AH. Posttraumatic headache: classification by symptom-based clinical profiles. Headache. (2018) 58:873–82. 10.1111/head.13311 29737529

[15] Finkel AG Ivins BJ Yerry JA Karic JS Scher A Choi YS. Which matters more? A retrospective cohort study of headache characteristics and diagnosis type in soldiers with mTBI/concussion. Headache. (2017) 57:719–28. 10.1111/head.13056 28239838

[16] Finkel AG Yerry JA Klaric JS Ivins BJ Scher A Choi YS. Headache in the military service members with a history of mild traumatic injury: a cohort study of diagnosis and classification. Cephalalgia. (2017) 37:548–59. 10.1177/0333102416651285 27206963

[17] Cohen SP Plunkett AR Wilkinson I Nguyen C Kurihara C Flagg A II. Headaches during war: analysis of presentation, treatment, and factors associated with outcome. Cephalalgia. (2012) 32:94–108. 10.1177/0333102411422382 21994113

[18] Bailie JM Remigio-Baker RA Cole WR McCulloch KL Ettenhofer ML West T. (2019). Use of the progressive return to activity guidelines may expedite symptom resolution after concussion for active duty military. Am J Sports Med. (2019) 47:3505–13. 10.1177/0363546519883259 31718246

[19] Hoffman JM Lucas S Kikmen S Braden CA Brown AW Brunner R. Natural history of headache after traumatic brain injury. J Neurotrauma. (2011) 28:1719–25. 10.1089/neu.2011.1914 21732765 PMC3172878

[20] Headache Classification Committee of the International Headache Society (IHS) The international classification of headache disorders, 3rd edition. Cephalalgia. (2018) 38:1–211. 10.1177/0333102417738202 29368949

[21] Cicerone K Kalmar K Persistent postconcussion syndrome. J Head Trauma Rehabil. (1995) 10:1–17. 10.1097/00001199-199510030-00002

[22] Meterko M Baker E Stolzmann KL Hendricks AM Cicerone KD Lew HL. Psychometric assessment of the neurobehavioral symptom inventory-22: the structure of persistent postconcussive symptoms following deployment-related mild traumatic brain injury among veterans. J Head Trauma Rehabil. (2012) 27:55–62. 10.1097/HTR.0b013e318230fb17 22190009

[23] Vanderploeg RD Silva MA Soble JR Curtis G Belanger HG Donnell AJ. The structure of post-concussion symptoms on the neurobehavioral symptom inventory: a comparison of alternative models. J Head Trauma Rehabil. (2015) 30:1–11. 10.1097/HTR.0000000000000009 24263177

[24] McKeon JMM Livingston SC Reed A Hosey RG Black WS Bush HM. Trends in concussion return-to-play timelines among high school athletes from 2007 through 2009. J Athl Train. (2013) 48:836–43. 10.4085/1062-6050-48.6.17 24143901 PMC3867096

[25] Makdissi M Darby D Maruff P Ugoni A Brukner P McCrory PR. Natural history of concussion in sport: markers of severity and implications for management. Am J Sports Med. (2010) 38:464–71. 10.1177/0363546509349491 20194953

[26] McCrea M Guskiewicz K RAndolph C Barr WB Hammeke TA Marshall SW. Effects of symptom-free waiting period on clinical outcome and risk of reinjury after sport-related concussion. Neurosurgery. (2009) 65:876–83. 10.1227/01.NEU.0000350155.89800.00 19834399

[27] McClincy MP Lovell MR Pardini J Collins MW Spore MK. Recovery from sports concussion in high school and collegiate athletes. Brain Injury. (2006) 20:33–39. 10.1080/02699050500309817 16403698

[28] McCrory P Meeuwisse WH Aubry M Cantu B Dvooak J Echemendia RJ. Consensus statement on concussion in sport: the 4th international conference on concussion in sport held in Zurich, November 2012. Br J Sports Med. (2013) 47:250–8. 10.1136/bjsports-2013-092313 23479479

[29] Remigio-Baker RA Kiser S Ferdosi H Gregory E Engel S Sebesta S. Current patterns of primary care provider practices for the treatment of post-traumatic headache in active duty military settings. PLoS ONE. (2020) 15:e0236562 10.1371/journal.pone.0236762 32706834 PMC7380628

[30] Misky GJ Wald HL Coleman EA. Post-hospitalization transitions: examining the effects of timing of primary care provider follow-up. J Hosp Med. (2010) 5:392–7. 10.1002/jhm.666 20578046

[31] Kontos AP Jorgensen-Wagers K Trbovich AM. Association of time since injury to the first clinic visit with recovery following concussion. JAMA Neurol. (2020) 77:435–40. 10.1001/jamaneurol.2019.4552 31904763 PMC6990755

[32] Ponsford J Willmott C Rothwell A Cameron P Kelly A-M Nelms R. Impact of early intervention on outcome following mild head injury in adults. J Neurol Neuros Psychiatry. (2002) 73:330–32. 10.1136/jnnp.73.3.330 12185174 PMC1738009

[33] Mac Donald CL Johnson AM Wierzechowski L Kassner E Stewart T Nelson EC. Outcome trends after US military concussive traumatic brain injury. J Neurotrauma. (2017) 34:2206–19. 10.1089/neu.2016.4434 27198861 PMC5510713

[34] Andelic N Howe EI Hellstrom T Sanchez MF Lu J Lovstad M. Disability and quality of life 20 years after traumatic brain injury. Brain Behav. (2018) 8:e01018. 10.1002/brb3.1018 29888869 PMC6043714

[35] O'Bryant SE Marcus DA Rains JC Penzien DB. The neuropsychology of recurrent headache. Headache. (2006) 46:1364–76. 10.1111/j.1526-4610.2006.00579.x 17040333

[36] Baskin SM Lipchick GL Smiterman TA. Mood and anxiety disorders in chronic headache. Headache. (2006) 46:76–87. 10.1111/j.1526-4610.2006.00559.x 17034402

[37] Brennan KC Charles A. Sleep and headache. Semin Neurol. (2009) 29:406–18. 10.1055/s-0029-1237113 19742415 PMC5605773

[38] Chan TLH Woldeamanuel YW. Exploring naturally occuring clinical subgroups of post-traumatic headache. J Headache Pain. (2020) 21:12. 10.1186/s10194-020-1080-2 32033526 PMC7006085

