# Supplementary material for: Provider Training in the Management of Headache Following Concussion Clinical Recommendation: Promoting a Standardized Means for Efficient Patient Recovery and Timely Return to Duty
Source: Front Neurol. 2020 Oct 15;11:559311. doi: 10.3389/fneur.2020.559311 (PMC7593659; doi:10.3389/fneur.2020.559311)
Supplement: Supplementary file 1 [file Data_Sheet_1.pdf]

SUPPLEMENTARY MATERIAL

Supplementary Table 1. List of questions during provider interview #2 (pre-CR training) and #3 (post-CR training)

Question Items	Question Asked	
	Pre-training	Post-training
**Knowledge of and Experience with PTH**		
*'… on average how many patients with headache following concussion do you treat per week?		
*Prior to the training you received with a DVBIC staff member on [DATE], were you familiar with the Management for Headache Following Concussion Clinical Recommendation?		X
*If yes: To what degree? … aware of the basic principles (or) …well-informed on the specific recommendations		X
*On a scale of 1 to 10, to what extent do you feel you understand the clinical recommendation Management of Headache Following Concussion? (1 is do not understand at all and 10 is completely understand).		X
*On a scale of 1 to 10, how comfortable are you providing care for patients with headache following concussion? (1 is not at all comfortable and 10 is completely comfortable).	X	X
*Can you describe some of the challenges in providing care for patients with headache following concussion?	X	X
*Please briefly describe the key principles in the Management of Headache Following Concussion Clinical Recommendation		X
*'… a scale of 1 to 10, how similar is the approach from one headache patient to the next? (1 is never the same and 10 is exactly the same)	X	X
*What factors contribute to differences in care you provide from one patient to the next?	X	X
*'…do you typically recommend any medical follow-up to patients?'	X	X
*How many follow-up visits with the patient do you recommend?'	X	X
*When do you recommend the visits occur (e.g., one day later)?'	X	X
*How do you refer patients to a rehabilitation provider and/or higher level of care?'	X	X
*To what type of provider would you typically refer a concussion patient with headache?'	X	X
*'…what factors determine when you refer a patient to a rehabilitation provider or higher level of care?'	X	X
*'…what factors determine when you consider a patient ready to return to duty?'	X	X
*'…have you distributed to patients the clinical recommendation's patient education materials?'	X	X
*'…have you verbally instruct patients according to the guidance in the materials?'	X	X
**Patient Change and Compliance with Treatment**		
*'Overall, do you think the Management of Headache Following Concussion Clinical Recommendation has the potential to improve patient outcomes?'		X
*In your view, how long (e.g., how many weeks, months) does it take to typically recover from concussion *regardless of medical care*?'	X	X
*In your view, how long (e.g., how many weeks, months) does it take for patients to recover *as a result of the care* you provide…?	X	X

Supplementary Table 2. Patient-reported headache (HA) history by intervention group (care as usual [CAU] vs. clinical recommendation [CR+] patient group)

Variables	Overall N=35	Intervention Group		
		CAU, n=21	CR+, n=14	p
**Did your HA start or worsen after your concussion? N (%)**				NA
Yes	35 (100.0)	21 (100.0)	14 (100.0)	0.172
Immediately/same day	24 (68.6)	12 (57.1)	12 (85.7)	
>1 day and w/in 1 wk	9 (25.7)	7 (33.3)	2 (14.3)	
>1 wk to 1 mo	2 (5.7)	2 (9.5)	0 (0.0)	
>1 mo	0 (0.0)	0 (0.0)	0 (0.0)	
No	0 (0.0)	0 (0.0)	0 (0.0)	
**Do you have continuous 24/7 HA? N (%)**				0.581
Yes	18 (51.4)	10 (47.6)	8 (57.1)	
No	17 (48.6)	11 (52.4)	6 (42.9)	
**About how often do you have headaches per week? Median (IQR),**	7 (4, 7)	7 (4, 7)	7 (3.5, 7)	0.607
**How long do your HA usually last if you do not treat them? Median (IQR)**				
Days (n=10)	2 (1, 7)	3.5 (1, 7)	2 (1, 13.5)	0.820
**How painful are your headaches usually? Median (IQR)[a]**	6 (5, 8)	6 (5, 9)	5 (5, 8)	0.682
**How painful is your headache at its worse? Median (IQR)[a]**	8 (7, 10)	8 (7, 10)	8.5 (8, 10)	0.285
**Is the pain usually on both sides of your head or just one side? N (%)**				0.673
Both sides	21 (60.0)	12 (57.1)	9 (64.3)	
One side	14 (40.0)	9 (42.9)	5 (35.7)	
**Is the pain usually in a particular location? N (%)**				0.324
Yes	30 (85.7)	19 (90.5)	11 (78.6)	
No	5 (14.3)	2 (9.5)	3 (21.4)	
**During the HA, does the pain throb or pulsate? N (%)**				0.805
Yes	32 (91.4)	19 (90.5)	13 (92.9)	
No	3 (8.6)	2 (.5)	1 (7.1)	
**During the HA, does physical activity make it worse? N (%)**				0.490
Yes	28 (80.0)	16 (76.2)	12 (85.7)	
No	7 (20.0)	5 (23.8)	2 (14.3)	
**During the HA, does light bother you more than normal? N (%)**				0.121
Yes	28 (80.0)	15 (71.4)	13 (92.9)	
No	7 (20.0)	6 (28.6)	1 (7.1)	
**During the HA, does noise bother you more than normal? N (%)**				0.298
Yes	24 (68.6)	13 (61.9)	11 (78.6)	
No	11 (31.4)	8 (38.1)	3 (21.4)	

Variables	Overall N=35	Intervention Group		
		CAU, n=21	CR+, n=14	p
**During the HA, are you sick to your stomach or do you vomit? N (%)**				0.072
Yes	16 (45.7)	7 (33.3)	9 (64.3)	
No	19 (54.3)	14 (66.7)	5 (35.7)	
**Do you ever see things like spots, starts, flashing lights, zig zag lines or loss of vision with your HA? N (%)**				0.040*
Yes	26 (74.3)	13 (61.9)	13 (92.9)	
No	9 (25.7)	8 (38.1)	1 (7.1)	
**Do the vision changes grow slowly over a period of minutes or do they begin all at once? N (%)**				0.308
Grow slowly	25 (96.2)	13 (100.0)	12 (92.3)	
All at once	1 (3.9)	0 (0.0)	1 (7.7)	
**During the HA, do you experience neck pain more than normal? N (%)**				0.890
Yes	18 (51.4)	11 (52.4)	7 (50.0)	
No	17 (48.6)	10 (47.6)	7 (50.0)	
**Since your concussion, what medications or treatments have you tried to help your HA? N (%)[b]**				NA
Acetaminophen	22 (62.9)	10 (47.6)	12 (85.7)	
NSAIDs	25 (71.4)	17 (81.0)	8 (57.1)	
Prescription medication(s)	12 (34.3)	6 (28.6)	6 (42.9)	
Non-pharmacological treatment	17 (48.6)	11 (52.4)	6 (42.9)	
Other	2 (5.7)	2 (9.5)	0 (0.0)	
**Have you tried a medication or treatment/therapy that initially worked, at least for a short period of time, but no longer works? N (%)**				0.656
Yes	11 (31.4)	6 (28.6)	5 (35.7)	
No	24 (68.6)	15 (71.4)	9 (64.3)	
**What helps your headaches the most at this time? N (%)**				0.796
Medication	10 (28.6)	6 (28.6)	4 (28.6)	
Specific therapy	0 (0.0)	0 (0.0)	0 (0.0)	
Relaxing activity	19 (54.3)	12 (57.1)	7 (50.0)	
Other	3 (8.6)	2 (9.5)	1 (7.1)	
Nothing helps	3 (8.6)	1 (4.8)	2 (14.3)	
**Did you suffer from HAs prior to your concussion? N (%)**				0.863
Yes	7 (20.0)	4 (19.1)	3 (21.4)	
No	28 (80.0)	17 (81.0)	11 (78.6)	

HA: Headache; IQR=interquartile range

[a] Based on a scale of 1 to 10 where 1 is least painful and 10 is most painful

[b] Multiple responses are reported by patient participants and, thus, values are not mutually exclusive. Percentages are based on total number of participants per column as the denominator (i.e., 35 for total, 21 for CAU and 14 for CR+).

*Significant p-value<0.05.

Supplementary Table 3. Patient-reported lifestyle (median [IQR]) measures by follow-up time and intervention group (care as usual [CAU]; clinical recommendation [CR+] patient group) by time

Lifestyle	Intervention Group		
	CAU, n=21	CR+, n=14	p
**Average number experienced in the last two weeks**			
**Cups of caffeinated drinks per day**			
T0 (<=72 hours post-enrollment)	2 (0, 3)	1 (0, 2)	0.161
T1 (1 week post-enrollment)	1 (0, 1.5)	1 (0, 2)	0.913
T2 (1 month post-enrollment)	1 (0, 2)	0.5 (0, 2)	0.757
Change from T0 to T1	0 (-1.3, 0.5)	0 (0, 0)	0.097
Change from T0 to T2	0 (-4.5, 1)	0 (0, 0)	0.240
**Hours of sleep per night**			
T0 (<=72 hours post-enrollment)	6 (5, 6)	5 (4, 6)	0.618
T1 (1 week post-enrollment)	5 (4, 6)	6 (5, 6)	0.331
T2 (1 month post-enrollment)	5 (5, 6)	5 (4.5, 6)	0.665
Change from T0 to T1	-0.5 (-1, 0.3)	0 (0, 1)	0.826
Change from T0 to T2	0 (-1, 2)	-0.5 (-1.5, 0.5)	0.133
**Cigarettes smoked per day**			
T0 (<=72 hours post-enrollment)	0 (0, 0)	0 (0, 0)	0.680
T1 (1 week post-enrollment)	0 (0, 0.1)	0 (0, 0)	0.386
T2 (1 month post-enrollment)	0 (0, 0)	0 (0, 0)	0.197
Change from T0 to T1	0 (0, 0)	0 (0, 0)	0.444
Change from T0 to T2	0 (0, 0)	0 (-0.5, 0)	0.317
**Alcoholic beverages per day**			
T0 (<=72 hours post-enrollment)	0 (0, 2)	0.5 (0, 3)	0.313
T1 (1 week post-enrollment)	0 (0, 0)	0.5 (0.4)	0.015*
T2 (1 month post-enrollment)	0 (0, 2)	0 (0, 0.25)	0.205
Change from T0 to T1	0 (-0.5, 0)	0 (0.0)	0.119
Change from T0 to T2	0 (0, 1)	-0.5 (-1.5, 0)	0.254
**Meals per day**			
T0 (<=72 hours post-enrollment)	2 (2, 3)	3 (2, 3)	0.589
T1 (1 week post-enrollment)	2.5 (2, 3)	3 (2, 3)	0.208
T2 (1 month post-enrollment)	2 (2, 3)	3 (2, 3)	0.145
Change from T0 to T1	0 (0, 0)	0 (0, 0)	0.356
Change from T0 to T2	0 (-0.5, 0)	0 (-0.5, 0)	0.163
**Glasses of water per day**			
T0 (<=72 hours post-enrollment)	10 (4, 16)	8 (5, 8)	0.285
T1 (1 week post-enrollment)	9 (6.5, 14)	10 (4, 16)	0.725
T2 (1 month post-enrollment)	9 (8, 16)	7 (4.5, 13)	0.199
Change from T0 to T1	0 (-1.8, 0)	2 (-2, 7)	**0.040***
Change from T0 to T2	0 (-2, 6)	-0.3 (-3, 2.5)	0.572
**Did your provider suggest that you take either over-the-counter or prescription medication? N (%)**			
Yes			
T0 (<=72 hours post-enrollment)	5 (23.8)	2 (14.3)	0.490
T1 (1 week post-enrollment)	5 (25.0)	1 (7.4)	0.179
T2 (1 month post-enrollment)	4 (26.7)	3 (25.0)	0.922
Change from T0 to T1	0 (0, 0)	0 (0, 0)	0.422

**Lifestyle**	**Intervention Group**		
	**CAU, n=21**	**CR+, n=14**	**p**
Change from T0 to T2	0 (0, 0)	0 (-0.5, 0)	0.722
**If yes, to what extend did/are you following your recommended HA management plan?**			
T0 (<=72 hours post-enrollment)	4.5 (4, 5)	4 (3, 5)	0.274
T1 (1 week post-enrollment)	4 (4, 5)	3.5 (2, 4)	0.060
T2 (1 month post-enrollment)	4 (4, 5)	4 (3, 5)	0.277
Change from T0 to T1	0 (0, 0)	0 (-1, 0)	0.654
Change from T0 to T2	0 (0, 0)	0 (0, 0.5)	0.553

*Significant p-value<0.05.

Supplementary Table 4. Patient-reported neurobehavioral symptoms (median [IQR]) by follow-up time and intervention group (care as usual [CAU] vs. clinical recommendation [CR+] patient group): total, by categories (cognitive, vestibular, somatosensory, affective) and by individual headache symptom

	Intervention Group		
	**CAU, n=21**	**CR+, n=14**	**p**
**Total symptoms**			
T0 (<=72 hours) (n=35)	41 (16, 45)	43.5 (28, 53)	0.288
T1 (1 week) (n=34)	27.5 (15, 47.5)	34.5 (26, 44)	0.353
T2 (1 month) (n=27)	20 (8, 45)	28.5 (21.5, 44.5)	0.660
Change from T0 to T1	-4.5 (-13.5, 4)	-7.5 (-12, 2)	0.820
Change from T0 to T2	-11 (-19, 2)	-14 (-23.5, -5)	0.366
**Cognitive Symptoms**			
T0 (<=72 hours) (n=35)	6 (2, 13)	8.5 (5, 13)	0.287
T1 (1 week) (n=34)	4 (1, 11)	7 (5, 10)	0.284
T2 (1 month) (n=27)	5 (1, 14)	5.5 (2, 10.5)	0.902
Change from T0 to T1	-0.5 (-3.5, 0)	-2 (-3, 2)	0.972
Change from T0 to T2	-1 (-2, 0)	-3 (-4.5, -1.5)	0.085
**Vestibular symptoms**			
T0 (<=72 hours) (n=35)	4 (3, 6)	4.5 (2, 7)	0.598
T1 (1 week) (n=34)	3 (2, 6.5)	4 (2, 6)	0.751
T2 (1 month) (n=27)	2 (1, 5)	1 (0, 4.5)	0.273
Change from T0 to T1	-0.5 (-3, 0.5)	-1 (-2, 1)	0.819
Change from T0 to T2	-1 (-3, 0)	-2.5 (-4, -1.5)	0.097
**Somatosensory symptoms**			
T0 (<=72 hours) (n=35)	9 (6, 16)	11.5 (9, 16)	0.303
T1 (1 week) (n=34)	7 (4, 13.5)	9.5 (9, 12)	0.240
T2 (1 month) (n=27)	5 (1, 9)	9 (7, 11.5)	0.123
Change from T0 to T1	-2 (-3.5, 1)	-2 (-4, 2)	0.930
Change from T0 to T2 (n=27)	-4 (-7, 1)	-5 (-7, -2)	0.641
**Affective symptoms**			
T0 (<=72 hours) (n=35)	10 (4, 16)	13 (5, (17)	0.345
T1 (1 week) (n=34)	10 (4.5, 14.5)	10.5 (7, 16)	0.318
T2 (1 month) (n=27)	10 (2, 15)	10 (6, 16.5)	0.714
Change from T0 to T1	-2 (-3, 1.5)	-1.5 (-4, 2)	0.902
Change from T0 to T2	-2 (-5, 3)	-2 (-4.5, 0.5)	0.538
**Headache Item Only**			
T0 (<=72 hours) (n=35)	3 (2, 4)	4 (3, 4)	0.069
T1 (1 week) (n=34)	3 (2, 4)	3 (3, 4)	0.237
T2 (1 month) (n=27)	2 (1, 4)	2.5 (2, 3.5)	0.454
Change from T0 to T1	0 (-1, 0)	0 (-1, 0)	0.684
Change from T0 to T2	-1 (-2, 0)	-1 (-1.5, 0)	0.764

IQR=interquartile range
